# Single-cell transcriptomic analysis reveals intra-tumoral heterogeneity and immunotherapy strategies in high-grade serous ovarian cancer

**DOI:** 10.1016/j.isci.2026.115266

**Published:** 2026-03-06

**Authors:** Chunhui Gao, Xiaogang Lv, Anqi Pan, Lipai Chen, Qianqian Liu, Donghui Zhang, Wenjuan Wu

**Affiliations:** 1 Department of Critical Care Medicine, Guangzhou Institute of Cancer Research, the Affiliated Cancer Hospital, Guangzhou Medical University, Guangzhou, Guangdong 510095, China; 2 Department of Gynecological Oncology, Guangzhou Institute of Cancer Research, the Affiliated Cancer Hospital, Guangzhou Medical University, Guangzhou, Guangdong 510095, China; 3 Department of Pathology, Guangzhou Institute of Cancer Research, the Affiliated Cancer Hospital, Guangzhou Medical University, Guangzhou, Guangdong 510095, China

**Keywords:** Cancer, Microenvironment, Transcriptomics

## Abstract

High-grade serous ovarian cancer (HGSOC) is the most common type of ovarian cancer, characterized by high intra-tumoral heterogeneity and platinum resistance. In this study, single-cell RNA sequencing data with HGSOC were analyzed. We identified seven major cell types in HGSOC and observed significant changes in the proportions of cancer-associated fibroblasts and epithelial cells as tumor drug resistance increased. The abundance of *IFIT1*^+^ and *CXCL10*^+^ epithelial cells highly expressing MHC class Ⅰ molecules, which are strongly associated with antigen processing and presentation, was found to improve patients’ outcomes. Ultimately, the relationships between *IFIT1*, MHC class Ⅰ molecules, and CD8^+^ T cells were verified in additional cohorts, using multiplex immunohistochemistry. Our data suggest that *IFIT1*^+^ epithelial cells might enhance immune surveillance via increasing the expression of MHC class Ⅰ molecules and activating CD8^+^ T cells. Our findings provide a valuable resource for deciphering intra-tumoral heterogeneity and mechanisms driving the aggressiveness of HGSOC, revealing potential biomarkers for anti-cancer treatment.

## Highlights

• A single-cell RNA-seq atlas reveals extensive intra-tumoral heterogeneity in HGSOC
• Tumor-associated fibroblasts and epithelial cells promote chemoresistance and metastasis
• *IFIT1*^+^ epithelial cells overexpressing MHC class Ⅰ are associated with good prognosis

## Graphical abstract

**Figure:**
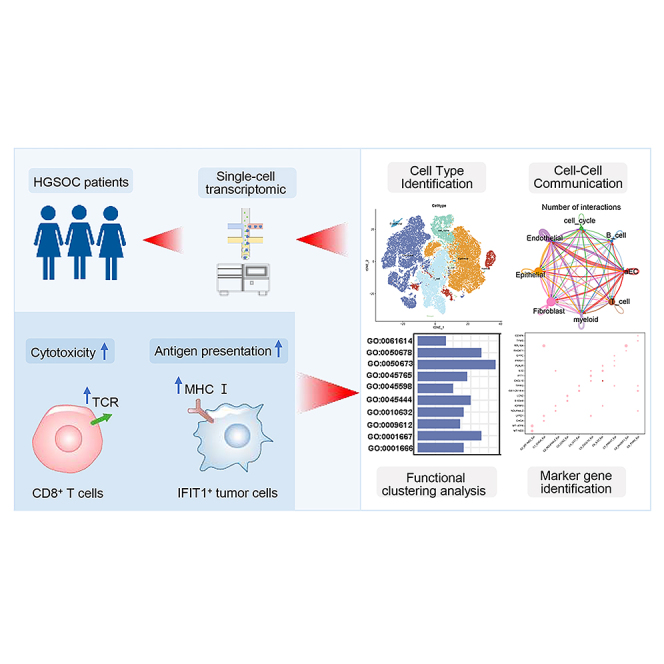

## Introduction

Epithelial ovarian cancer (EOC) is the most fatal type of gynecological cancer.^1^ Due to its asymptomatic nature in early stages, more than 80% of EOC cases are diagnosed at advanced stage (Ⅲ or Ⅳ) with peritoneal metastasis.^2^^,^^3^ The 5-year survival rate of EOC is <30%.^4^ High-grade serous ovarian cancer (HGSOC) is initially highly sensitive to chemotherapy.^5^^,^^6^ However, an estimated 85% of patients with EOC who achieve remission following first-line chemotherapy eventually experience recurrence and develop drug resistance.^7^ The origins of HGSOC recurrence and chemoresistance remain elusive due to high intra-tumoral heterogeneity. A better understanding of the complex cellular and molecular characteristics of the tumor microenvironment (TME) that distinguish metastatic and resistant HGSOCs will provide valuable insights into its regulatory mechanisms. This knowledge could facilitate the development of novel immunotherapeutic strategies for HGSOCs.

HGSOC is a copy number-driven cancer that has exceptionally high intra-tumoral heterogeneity and almost 100% prevalence of TP53 mutations,^8^^,^^9^ which impedes overcoming platinum resistance. Bulk RNA profiling has been used to categorize HGSOC into molecular subtypes.^10^ However, bulk RNA sequencing (RNA-seq) averages gene expression and may omit rare populations that may drive disease development and progression. Moreover, bulk expression data represent the collective reflection of signals from the tumor and TME, the latter of which can be a strong driver of tumor invasion. Single-cell RNA-seq (scRNA-seq) has emerged as a powerful tool to interrogate tumor composition, revealing cellular heterogeneity and gene regulatory networks at single-cell resolution. Single-cell transcriptomics can stratify metastatic ovarian cancer based on tumor-infiltration patterns of T cell subsets, thereby predicting responses to immunotherapies.^11^

In this study, we analyzed the single-cell transcriptomic profiles of HGSOC and characterized the heterogeneity among different cell types from multiple dimensions. We identified that *CXCL10*^+^ and *IFIT1*^+^ epithelial cells are linked to antigen processing and presentation and are the key predictors of favorable patient outcomes. Furthermore, it was found that *IFIT1*^+^ epithelial cells enhance anti-tumor immunity by promoting MHC class Ⅰ expression and activating CD8^+^ effector T cells. By delineating the transcriptomic heterogeneity in HGSOC, our work deepens the understanding of mechanisms driving its progression while facilitating the discovery of novel prognostic biomarkers and therapeutic targets.

## Results

### TME of HGSOC at single-cell resolution

**Figure 1:**
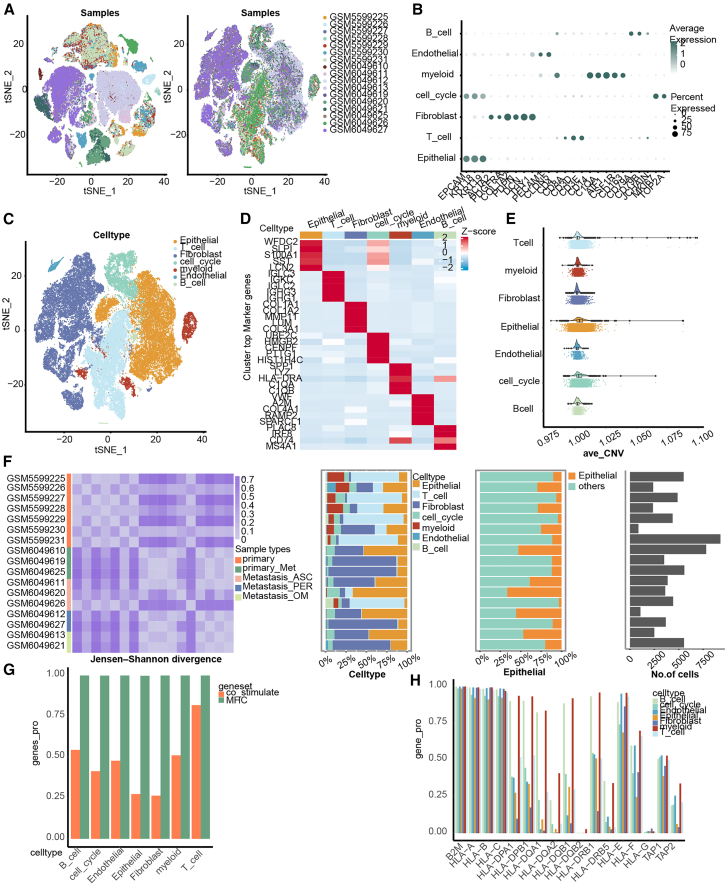
Single-cell transcriptome profiles of HGSOC (A) Comparison of t-SNE dimensional reduction plots before and after batch effect correction, with different colors representing different samples. (B) Bubble plot showing the expression levels of classic markers for major cell types, with darker colors indicating higher gene expression levels and larger dots representing a greater proportion of gene expression in the cells. (C) t-SNE dimensional reduction plots of the seven identified major cell types, with different colors representing different cell types. (D) Heatmap of the top 5 expressed genes in cell clusters. (E) Mean gene copy numbers in different cells, with different colors indicating different cell types. (F) JS divergence between different samples, cell proportions, proportions of epithelial cells, and number of cells included in each sample. (G) Overall proportions of co-stimulatory molecules and MHC molecules in different cells. (H) Proportions of MHC molecules in different cells. JS divergence, Jensen-Shannon divergence.

To investigate HGSOC heterogeneity at single-cell resolution, we collected 17 (10 primary, 3 ascites metastasis, 2 peritoneal metastasis, and 2 omental metastasis) tissue samples from 10 patients with HGSOC (Tables S2 and S3). After quality control (QC) procedures, we obtained transcriptomic profiles for 71,176 cells (Figure S1A). To eliminate potential biases resulting from combining different datasets, we performed batch correction between samples (Figure 1A). Applying the unsupervised shared nearest neighbor method, a total of 27 cell clusters (Figure 1B) were identified and annotated, based on canonical cell marker genes, to seven major cell type-specific clusters (Figure 1C): epithelial cell cluster, T cell cluster, fibroblast cell cluster, cell cycle cluster, myeloid cell cluster, endothelial cell cluster, and B cell cluster. To investigate the compositional heterogeneity between these major cell types, we employed the “FindAllMarkers” function to identify differentially expressed genes (DEGs). The heatmap in Figure 1D shows that there is almost no overlap between DEGs among the different cell types. Copy number variation (CNV) analysis (Figure 1E) revealed, in turn, significantly higher gene copy numbers in epithelial cells compared with those in other cell types. The Jensen-Shannon divergence (JSD) algorithm was next used to measure dissimilarity among the samples. The analysis revealed significant differences in the cell composition between samples that had metastasized and those that had not (Figure 1F). In particular, the proportion of T cells was significantly higher in non-metastatic samples, while fibroblasts and epithelial cells were more prevalent in metastatic samples and their corresponding primary sites. Subsequently, the proportions of MHC molecules and T cell co-stimulatory molecules based on literature were investigated in the major cell types (Figures 1G, 1H, and S2A). We found that co-stimulatory molecules, which are closely associated with T cells, exhibited higher expression levels in these cells. MHC class Ⅰ and other MHC molecules predominantly expressed in nearly all cell types. MHC class Ⅱ molecules primarily expressed in B cells and myeloid cells. These findings collectively confirm the significant heterogeneity observed among different cell types.

### Analysis of epithelial cell clusters in HGSOC

**Figure 2:**
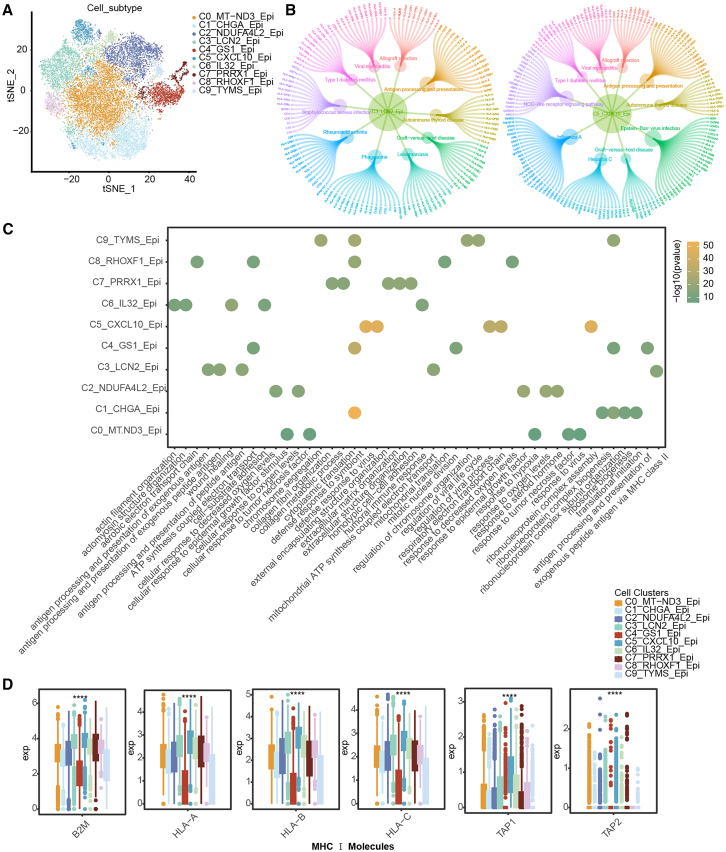
Analysis of epithelial cell subpopulations in HGSOC (A) t-SNE representation of epithelial cell subpopulations based on clustering, with different colors representing different cell subpopulations. (B) Top 10 pathways of subpopulations C_3 and C_5 by KEGG pathway enrichment analysis. (C) Bubble plot of GO functional annotation analysis. (D) Expression level of MHC class Ⅰ molecules in different cell clusters.

**Figure 3:**
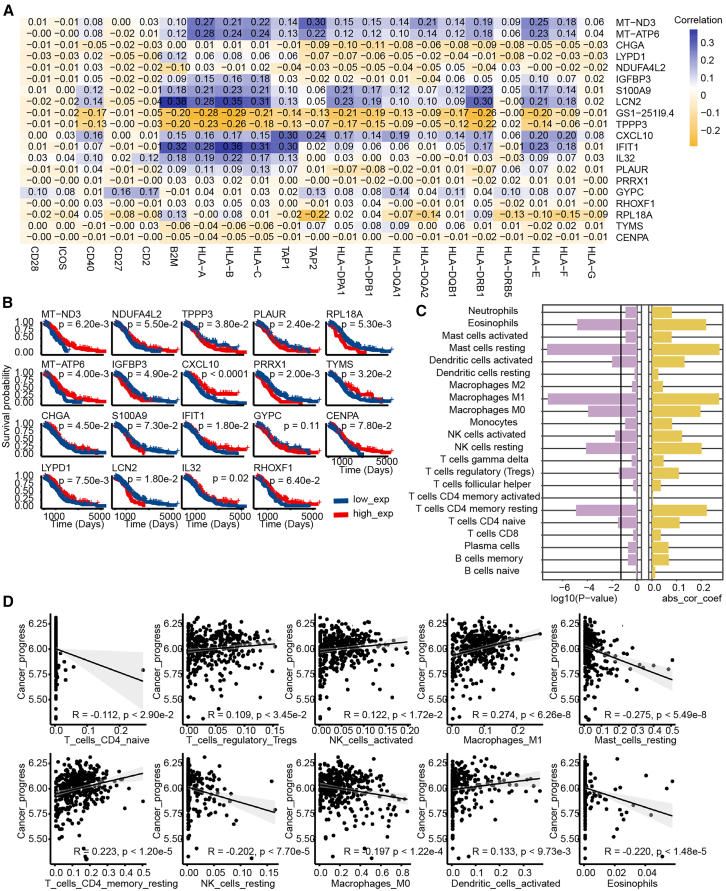
Correlation and survival analyses (A) Heatmap of the correlation coefficients between differentially expressed functional genes and the top two marker genes across cell clusters, with darker colors indicating stronger correlations. (B) Relationship between the top 2 differential genes in each epithelial cell cluster and patient prognosis in the TCGA bulk dataset. (C) Correlation coefficients and *p* values for the infiltration levels of 22 immune cell types and tumor progression activity. (D) Scatterplots of significantly correlated cell pairs, where each point represents a sample.

We next examined the transcriptional states of HGSOC-associated epithelial cells through graph-based clustering analysis. For the 10 clusters thus identified, we computed signatures based on the differentially upregulated genes between each of them and the other clusters (Figure 2A). Then, Kyoto encyclopedia of genes and genomes (KEGG) pathway and GO_BP enrichment analyses were conducted. The results revealed that distinct pathways and functions are associated with various cell clusters (Figures 2B, 2C, and S1B). The top 10 pathways enriched in clusters C3 and C5 displayed greater heterogeneity, except for antigen processing and presentation. This phenomenon was also observed in other clusters, highlighting the need for further in-depth investigation. Subsequently, we performed an immunogenicity analysis on the different epithelial cell clusters by assessing differential expression of co-stimulatory molecules and MHC molecules across them (Figures 2D, S2D, and S2E). The results indicated significant differences in the expression of relevant genes, except for *HLA-DQB2*, across all clusters. Therefore, the correlations between those genes (excluding *HLA-DQB2*) and the top two DEGs in each cluster were further analyzed. A heatmap was used to visualize the correlation coefficients between different genes (Figure 3A and Table S4). To better visualize gene correlations, the gene pairs with the highest (positive correlation) and lowest (negative correlation) correlation coefficients were further selected for scatterplot analysis (Figures S2B and S2C). Correlation analysis showed that the top two DEGs among the clusters were significantly correlated with different functional molecules. The prognostic significance of the DEGs was next analyzed based on TCGA bulk expression data. The expression levels of 19 matched DEGs were extracted, and the “surv_cutpoint” function from the “survminer” package was used to determine the optimal cutoff value for each gene. Then, patients were divided into high expression and low expression groups, and Kaplan-Meier survival curves were generated to visualized the DEGs’ prognostic significance (Figure 3B). We found that most DEGs were significantly associated with patient prognosis (log-rank test, *p* < 0.05).

Table S1. Participant characteristics, related to Figures 8G–8K

To further investigate the roles of different epithelial cell clusters in cancer, we downloaded the “pathways_in_cancer” pathway from the KEGG database. Additionally, ovarian cancer driver genes from the Network of Cancer Genes database^12^ were retrieved to annotate cell clusters associated with cancer progression. The “cell_cycle” pathway and genes such as *CDC20*, *CCNB1*, *CENPF*, and *PTTG1* were used to identify clusters related to the cell cycle. Through hypergeometric enrichment analysis, clusters C_0, C_2, C_5, C_6, and C_7 were significantly associated with ovarian cancer progression, while clusters C_4 and C_9 were significantly linked to the cell cycle (Table S5). Based on these annotations, we further analyzed the association between tumor progression-related epithelial cell and immune cell infiltration, using TCGA bulk expression data. First, DEGs in the tumor progression clusters were extracted from the single-cell annotation results. Subsequently, the activity of the tumor progression cell cluster in the TCGA bulk expression data was calculated using ssGSEA. Then, the correlation between the activity values of tumor progression-related cell clusters and the infiltration levels of 22 immune cells was analyzed (Figures 3C and 3D). The results revealed that 10 immune cell types had significant correlations with the activity of the tumor progression cell cluster. For example, functionally contrasting immune cell types, such as “NK cells activated” and “NK cells resting,” exhibited opposing states. This suggests that the tumor progression cluster of epithelial cells interacts with various immune cells, influencing HGSOC development and progression either by restriction or promotion of tumor cell growth.

Table S4. The correlation *p* values between differentially expressed functional genes and the top 2 marker genes across cell clusters, related to Figure 3A

### Heterogeneity of tumor-associated macrophages

**Figure 4:**
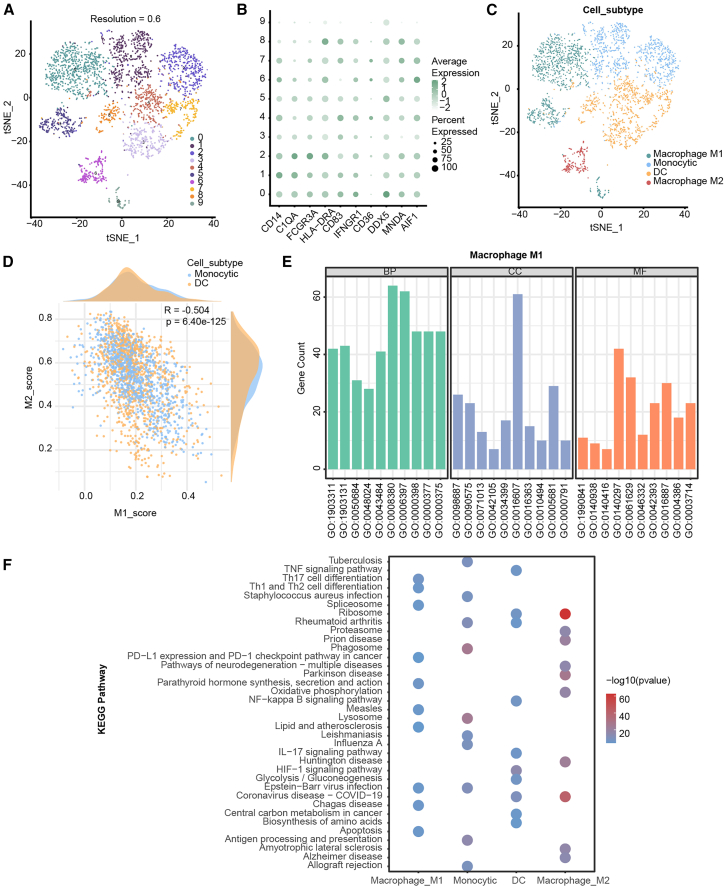
Analysis of tumor-associated macrophage heterogeneity (A) t-SNE representation of myeloid cells based on clustering, with different colors representing different cell clusters. (B) Bubble plot of the expression levels and proportions of classic marker genes in different cell clusters. (C) t-SNE plot of cell subpopulation annotation, with colors representing different cell subpopulations. (D) Correlation analysis between M1 and M2 macrophages, with each point representing a cell. (E) GO annotation for M1 macrophages. (F) Enriched KEGG pathways for different cell subpopulations.

Immune cells in the TME play complex roles in tumor progression. To explore the composition and abundance of infiltrating immune cells in HGSOC, we processed scRNA-seq data and identified through expression-based clustering analysis 10 immune cell clusters (Figure 4A). Three distinct myeloid populations, i.e., macrophages, monocytes, and dendritic cells (DCs), were further defined by comparing the abundance of canonical marker genes on sorted clusters (Figure 4B). Macrophages were further subdivided into two subtypes, M1 and M2 (Figure 4C). We scored all myeloid cells based on the upregulated DEGs from the M1 and M2 clusters. Our analysis revealed a strong positive correlation between these clusters (Figure 4D), which is consistent with the findings of recent studies showing co-expression of M1 and M2 markers in human cancers.^13^ Pathway enrichment and functional annotation analyses showed strong heterogeneity between different cell subpopulations, even between M1 and M2 macrophages (Figures 4E, 4F, and S3). For instance, pathways like apoptosis and Th1 and Th2 cell differentiation were significantly enriched in M1 macrophages but not in M2 macrophages. These findings show that even cell subpopulations with strong expression correlations can have significant functional differences.

### T cell profiling

**Figure 5:**
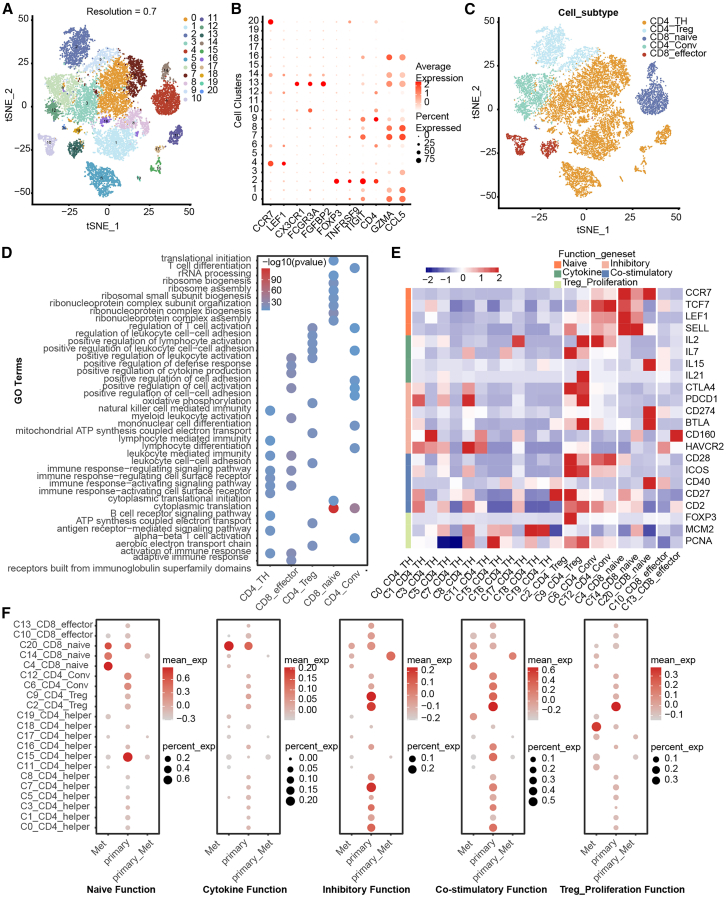
Expression profile of T cells (A) t-SNE plot of T cells based on clustering, with different colors representing different cell clusters. (B) Bubble plot of classic markers for different T cell subpopulations. (C) t-SNE plot of T cell annotation, with different colors representing different cell subpopulations. (D) Functional annotation for different T cell subpopulations. (E) Expression levels of different functional genes in various T cell clusters. (F) Expression levels of different functional gene sets in different samples.

T cells could be subtyped into 21 cell clusters according to marker gene expression (Figures 5A–5C, and S4). Then, annotation for biological function was conducted on 5 cell subpopulations, with results suggesting significant heterogeneity among them (Figure 5D). For example, CD4^+^ helper T cells were mainly enriched in activation of immune response and immune response-activating signaling pathway, suggesting their key role in the immune system. On the other hand, CD8_naive cells were primarily enriched in rRNA processing and ribosome assembly. The metabolic demands of CD8_naive T cells are increased when they are activated by antigens. To meet this demand, these cells need to rapidly produce various effector molecules, such as cytokines, enzymes, and receptors, a process that depends on the high activity of ribosomes. Next, we explored the expression levels of T cell-related functional genes in the different cell clusters (Figure 5E). We found that genes related to naive and cytokine functions were more highly expressed in CD4_conv and CD8_naive clusters, whereas the CD4_Treg subpopulation exhibited high expression of all functional genes except those related to the naive function. Additionally, we investigated the expression of these gene sets in different samples to identify potential dysfunctional T cell clusters (Figure 5F). The results indicated that in different functional gene sets, the high-expressing cell clusters also vary accordingly. This suggests that distinct functional genes may contribute to dysfunction in different cell subpopulations.

### Heterogeneity of cancer-associated fibroblasts and endothelial cells

**Figure 6:**
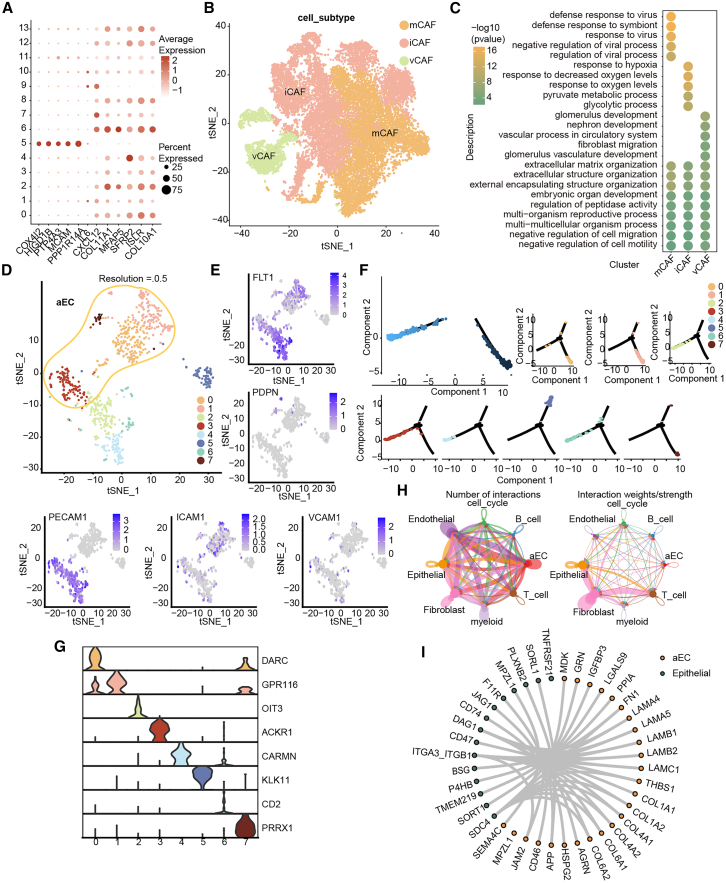
Heterogeneity of CAFs and endothelial cells (A) Expression levels of classic marker genes for fibroblasts in different cell clusters. (B) t-SNE plot of annotated fibroblast subpopulations, with different colors representing different CAF subpopulations. (C) Functional annotation of fibroblast subpopulations. (D) t-SNE of endothelial cell clusters based on clustering. (E) t-SNE plot of classic lymphatic and vascular marker genes. (F) Pseudotime trajectory analysis of different endothelial cell clusters. (G) Violin plot of the top DEG for each cell cluster, showing expression levels of different genes across different clusters. (H) Cell-cell interaction analysis. The left plot indicates the number of interactions between different cells, and the right plot indicates the strength of interactions. (I) Interaction between aECs and epithelial cells. CAFs, cancer-associated fibroblasts; aECs, activated endothelial cells.

We also extracted cancer-associated fibroblasts (CAFs) and re-clustered them using graph-based methods. Fourteen distinct cell clusters were thus identified. The expression levels and distribution of different marker genes were analyzed (Figures 6A and S5A). We found that *ISLR* expressed in almost all cell clusters, whereas genes like *COX4I2* expressed only in specific clusters. As a result, we annotated the fibroblasts into 3 subpopulations: vascular CAFs (vCAFs), inflammatory CAFs (iCAFs), and matrix CAFs (mCAFs) (Figure 6B). For each CAF subtype, we performed gene function annotation using the top 150 upregulated DEGs (if fewer than 150 genes were upregulated in a given cell subtype, all DEGs were used for enrichment analysis). The results indicated strong heterogeneity among the three subpopulations (Figure 6C). However, some common functions, such as extracellular matrix organization and extracellular structure organization, were also identified.

Next, we extracted all endothelial cells and further identified subpopulations through re-clustering. Using unsupervised clustering, all endothelial cells were divided into eight clusters, and the specific genes for each cluster were identified (Figures 6D and S5B). The expression of lymphatic and vascular marker genes (*FLT1*, *PDPN*, and *PECAM1*) was analyzed in different subpopulations. We found that *FLT1* was uniformly expressed in almost all endothelial cells, while *PECAM1* was less expressed, and *PDPN* expression was almost absent. This indicates that the endothelial cells in our dataset were derived from blood vessels rather than lymphatic vessels. Based on the expression levels of classic markers for activated endothelial cells, namely *ICAM1* and *VCAM1*, we classified clusters 0, 1, 3, and 7 as activated endothelial cells (aECs) (Figure 6E). Through pseudotime trajectory analysis, we observed two distinct cell fates, with one branch progressing from clusters 0, 1, and 7 toward cluster 5, and the other branch progressing toward clusters 2, 3, 4, and 6 (Figure 6F). In the analysis of the most significant DEGs in each cluster, we indeed found that the expression patterns of clusters 0 and 1 were more alike (Figure 6G).

Subsequently, we analyzed cell-cell interactions. Interactions occurred between various types of cells, although with varied intensity (Figure 6H). Since epithelial cells were identified as being more important in HGSOC in the previous analysis, we focused on the interactions between aECs and epithelial cells (Figure 6I). We found that among specific cell pairings, *DAG1*-, *ITGA3*-, and *SDC4*-expressing epithelial cells, were more likely to interact with aECs.

### Identification of cell types promoting tumor invasion in drug-resistant/metastatic HGSOC

**Figure 7:**
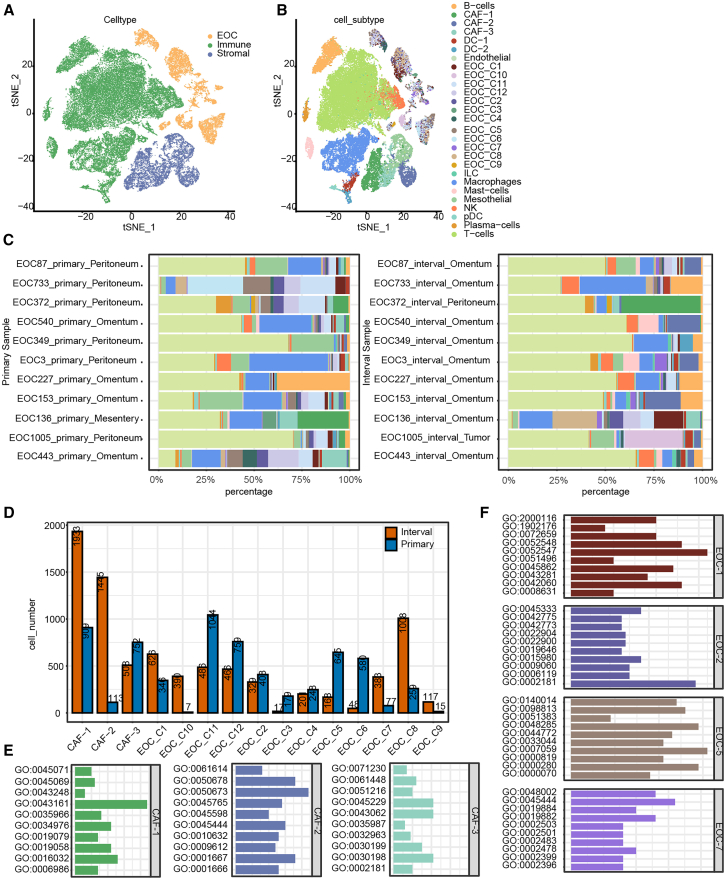
Identification of cell types promoting cancer cell invasion in drug-resistant/metastatic HGSOC (A) t-SNE plot of major cell types, with different colors representing different cell types. (B) t-SNE plot of cell subtypes, with different colors representing different cell subpopulations. (C) Cell proportion diagrams in different samples: the left plot represents primary samples, while the right plot represents metastatic/treated samples. (D) Number of cells in different subclusters of fibroblasts and epithelial cells. (E) Functional clustering analysis of different subclusters of fibroblasts. (F) Functional clustering analysis of different subclusters of epithelial cells.

We obtained ovarian cancer scRNA-seq data from the GSE165897 dataset, comprising 11 primary tumor samples and their corresponding metastatic sites. This dataset is annotated for cell types and subpopulations. Additionally, it includes a chemotherapy response score (CRS), ranging from 1 point (no or low tumor response) to 3 points (a complete or near-complete response), for each sample following post-neoadjuvant chemotherapy treatment. We visualized the data according to the source literature to enhance the observation of cell distribution (Figures 7A and 7B). Subsequently, we focused on the proportion of different cell subpopulations in the primary and metastatic sites. The samples were ordered by the increasing CRS scores. We observed that as tumor resistance increased (reflecting reduced or no treatment), the proportion of CAFs also increased in the samples. However, this phenomenon appears to be influenced by the site of metastasis (Figure 7C). Further analysis annotated CAFs to three subpopulations (Figure 7D). The analysis of samples with consistent tissue specificity revealed that after treatment, the proportions of CAF_1 and CAF_C3 subpopulations decreased, while the proportion of the CAF_2 subpopulation increased, suggesting an improved patient response to chemotherapy. Analysis of biological processes indicated that the CAF_2 fibroblast subpopulation was primarily enriched in pathways related to hypoxia response, epithelial cell proliferation and migration regulation, fat cell differentiation, and angiogenesis regulation. The CAF_1 fibroblast subpopulation was, in turn, found to be mainly enriched in proteasome-mediated ubiquitin-dependent protein catabolic process, cellular response to topologically incorrect protein, and response to endoplasmic reticulum stress. The CAF_3 subpopulation showed significant enrichment in pathways involved in the organization of the extracellular matrix, as well as in external encapsulating structure organization and extracellular structure organization (Figure 7E).

Based on our previous analyses, which suggested that EOCs play a critical role in HGSOC, we conducted subpopulation analysis of EOCs, identifying a total of 12 cell subpopulations (Figure 7D). Proportional analysis of subpopulations in samples with consistent tissue specificity before and after treatment revealed significant changes in the EOC_C1, EOC_C2, EOC_C5, and EOC_C7 subpopulations of EOCs. Moreover, patients who exhibited a greater reduction in these four subpopulations after treatment demonstrated a better response to chemotherapy. Further functional annotation revealed that the EOC_C1 subpopulation cells were primarily associated with regulation of endopeptidase and peptidase activity, wound healing, and intrinsic apoptotic signaling pathway in response to oxidative stress; EOC_C2 subpopulation cells were mainly enriched in cytoplasmic translation, cellular respiration, and energy derivation by oxidation of organic compounds; EOC_C5 cells were predominantly enriched in chromosome segregation, kinetochore organization, and nuclear division; and the EOC_C7 cells were primarily enriched in fat cell differentiation, antigen processing and presentation of peptide antigen, and antigen processing and presentation (Figure 7F).

### Identification of tumor marker-specific expression based on scRNA-seq

**Figure 8:**
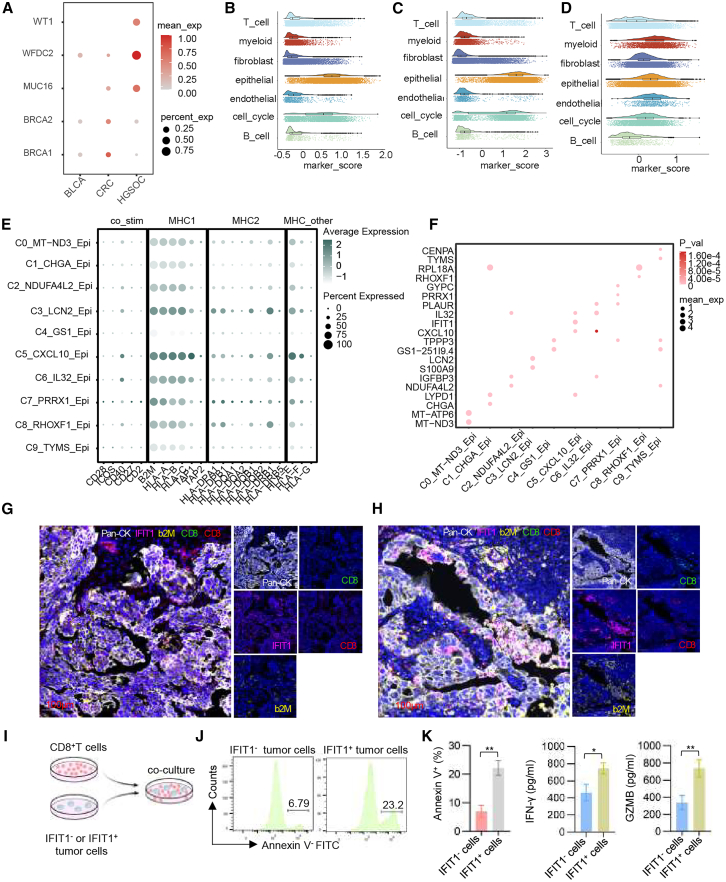
Validation of tumor-associated marker genes (A) Expression of tumor-associated markers in epithelial cells from different cancers. (B) Scoring for major cell types based on tumor-associated markers. (C) Scoring of tumor diagnostic marker-related genes for major cell types. (D) Scoring of EV marker genes for major cell types. (E) Expression levels of co-stimulatory molecules and MHC molecules in different epithelial cell clusters. (F) Expression levels of top 2 genes in different epithelial cell clusters. (G and H) mIHC images displaying the expression of *IFIT1*, epithelial marker (pan-CK), MHC class Ⅰ marker (B2M), and CD8^+^ T cell markers (CD3^+^/CD8^+^) in HGSOC patients with (G) unfavorable prognosis and (H) favorable prognosis; *n* = 50. (I–K) CD8^+^ T cells (effectors) were incubated with IFIT1^+^ or IFIT1^−^ tumor cells (targets) at a 10:1 effector-to-target (E:T) ratio for 72 h. (I) Schematic of the *in vitro* culture model. (J) Representative flow cytometry plots of the results of the apoptosis assays and the quantification of apoptosis assays based on their Annexin V^+^ percentages. (K) Levels of IFN-γ and granzyme B in the supernatant, measured via ELISA. Data are presented as the mean ± SEM, *n* = 5 pairs, including 5 patients and 5 healthy volunteers (J and K). ∗*p* < 0.05, ∗∗*p* < 0.01, two-tailed Student’s *t* test.

Previous studies have identified several clinically relevant molecular diagnostic markers for HGSOC, including *MUC16*, *WFDC2*, *BRCA1*, *BRCA2*, and *WT1*. To investigate whether HGSOC marker genes are exclusively expressed in epithelial cells in HGSOC, we analyzed scRNA transcriptome data from two other epithelial cancer types in the GEO database, i.e., colorectal cancer (GSE146771) and bladder cancer (GSE130001). The results indicated that HGSOC marker genes are also expressed in colorectal and bladder cancer cells (Figure 8A). This suggests that high expression of common tumor marker genes may be a shared feature of cancerous epithelial cells. Based on these markers, we employed AddModuleScore from the Seurat package to score all cells and analyze the expression levels of these genes across various cell types and clusters. The results showed that different cell types and clusters exhibited distinct expression levels of the marker genes (Figures 8B and S6). Compared with other cell types, epithelial cells demonstrated the highest score, highlighting a closer relationship with the diagnostic markers of HGSOC. Additionally, we performed a correlation analysis to identify genes significantly correlated with diagnostic markers among the top 10 DEGs between different cell types, using a correlation coefficient >0.7 and a *p* value <0.05 as thresholds. Next, marker genes for extracellular vesicle (EV) were obtained from the literature (Table S6). Based on the gene sets correlated with EV marker genes, we scored all cells and identified the cell types associated with these markers. The results revealed that epithelial cells ranked as the top cell type (Figures 8C and 8D). Subsequently, the expression levels of MHC and T cell co-stimulatory molecules based on literature were investigated in epithelial cells (Figure 8E). We found that cluster C5, which is closely associated with antigen processing and presentation, exhibited higher expression levels of MHC class Ⅰ molecules. Meanwhile, the top 2 genes of epithelial cells were visualized (Figure 8F). *IFIT1* predominantly expressed in cluster C5. High abundance of tumor cells expressing *IFIT1* was associated with a trend for better prognosis (Figure 3B). These findings collectively confirm that the expression of *IFIT1* in epithelial cells may have an important connection with MHC class Ⅰ molecules.

To validate our findings and examine the association between *IFIT1* and the TME, multiplex immunohistochemistry (mIHC) was performed. Samples with increased *IFIT1*-expressing cells demonstrated elevated expression levels of the epithelial marker pan-CK+ (Table S1). Moreover, these samples exhibited higher intensities of MHC class Ⅰ molecules (B2M^+^) and CD8^+^ T cell markers (CD3^+^/CD8^+^) (Figures 8G and 8H). Next, CD8^+^ T cell activation and killing assays were performed. *IFIT1*^+^ or *IFIT1*^−^ tumor cells were co-cultured with HLA-matched CD8^+^ T cells (Figure 8I). Compared with *IFIT1*^−^ tumor cells, the *IFIT1*^+^ tumor cells induced by CD8^+^ T cells showed increased apoptosis (Figures 8J and S7), ELISA examining IFN-γ and granzyme B revealed that *IFIT1*^+^ tumor cells significantly increased the secretion of these immune factors in the supernatants of CD8^+^ T cells (Figure 8K). These results confirmed that elevated *IFIT1* expression in epithelial cells potentially enhances MHC class Ⅰ molecule presentation, subsequently activating CD8^+^ T cell infiltration, thereby modulating the local immune microenvironment.

## Discussion

Ovarian cancer is known for its heterogeneity, manifested by genetic and phenotypic variations among tumor cells, which often lead to differences in treatment responses. This heterogeneity poses major treatment challenges and is closely linked to drug resistance.^14^ HGSOC is the most common subtype of ovarian cancer, accounting for approximately 80% of all ovarian cancer deaths.^15^ Thus, exploring the bases of such heterogeneity is pivotal for the prognostic improvement of patients with HGSOC. In this study, our analysis of single-cell transcriptome data from 17 primary and metastatic HGSOC tissue samples from 10 HGSOC patients showed that there were significant differences in the proportions of epithelial and fibroblast cell subpopulations before and after treatment and that these differences were tissue specific. Functional clustering analysis of epithelial cells revealed four subpopulations, termed C_1, C_2, C_5, and C_7, as the most active ones before and after treatment. Patients with a significant decrease in the proportion of these four clusters of cells after treatment showed better responses to anti-tumor therapy. Functions associated with these subpopulations include oxidative stress, cell metabolism, apoptosis, cell cycle regulation, and TME, all of which are classic mechanisms of platinum resistance in ovarian cancer.^16^

The different cell types in the HGSOC TME work together to perform essential functions, which is a complex biological process. Xu et al. used the transcriptomic results obtained through scRNA-seq analysis to depict the ecosystem landscape of HGSOC in its early or late stages.^17^ We established a comprehensive gene expression atlas of primary and metastatic samples in HGSOC and characterized their gene expression profiles based on scRNA-seq analysis. Further composition-based analysis between primary and metastatic samples demonstrated that the proportion of T cells was significantly increased, accompanied by a high expression of co-stimulatory molecules, in non-metastatic samples. Meanwhile, in metastatic samples and their corresponding primary foci, the proportion of fibroblasts and epithelial cells was significantly higher than in the other cell types. These findings indicate that significant heterogeneity exists between different samples and among different cell types.

CAFs are regarded as a homogeneous group within the TME. However, advancements of single-cell transcriptomics have enabled researchers to analyze the heterogeneity of CAFs in various cancer types.^18^^,^^19^^,^^20^ We profiled HGSOC-associated fibroblasts and noted that a post-treatment increase in the CAF_2 subpopulation, along with a decrease in CAF_1 and CAF_3 subpopulations, correlated with better response to chemotherapy. Functional clustering analysis suggested that these changes may be related to extracellular matrix organization and epithelial cell proliferation. CAFs in the TME induce a shift to a more fibrotic environment by changing the mechanical properties of the extracellular matrix, thereby affecting the efficacy of anti-tumor therapy.^21^ In addition, CAFs induce chemoresistance through other mechanisms, such as the secretion of EVs or the regulation of Wnt and Notch signaling pathways.^22^ Thus, we propose that certain subclusters of epithelial cells and fibroblasts are closely linked to the development of drug-resistant HGSOC.

Sandro Santagata’s team employed an integrated high-plex imaging and spatial transcriptomics approach to delineate the molecular evolution of HGSOC from precursor lesions to malignancy, and they confirmed that early HGSOC development is characterized by sustained IFN pathway activation.^18^^,^^23^ This manifests through coordinated upregulation of IFN-inducing factors, transcriptional regulators, and downstream effector genes, culminating in elevated MHC class Ⅰ expression that enhances tumor immunogenicity.^24^ Further analysis of the immunogenicity of each epithelial cell subgroup showed that the C5_(*CXCL10* and *IFIT1*) subpopulations had the closest relationship with MHC class Ⅰ molecules. KEGG-based clustering analysis indicated that C5 (*CXCL10* and *IFIT1*) subpopulations were associated with the antigen processing and presentation pathway. Prognostic analysis based on TCGA bulk expression data indicated that *IFIT1*, *CXCL10*, *LCN2*, and *S100A9* were significantly correlated with prognosis. Collectively, this mechanistic rationale prompted our investigation into the functional contributions of key IFN pathway genes (*CXCL10* and *IFIT1*) to HGSOC pathogenesis.^21^^,^^25^ *CXCL10*, a chemokine involved in immune surveillance, plays a pivotal role in attracting immune cells to the tumor site.^26^^,^^27^ Elevated expression of *CXCL10* in cancers suggests an active engagement with the immune system, possibly facilitating tumor recognition and response. However, this interaction is dual faceted. While *CXCL10* can attract immune cells to the tumor site, it may also contribute to immune evasion strategies deployed by cancer cells; therefore, its role in cancer biology is complex.^19^ The role of IFIT1 has been less reported in carcinoma. However, in the context of the innate immune response, John et al. described its function as a regulatory factor that mediates the mutual regulation of pro-inflammatory and interferon genes induced by bacteria.^28^ *IFIT1* interacts with *ANXA2* to enhance the cycling of *p*-EGFR and its downstream signaling, thereby driving the progression and metastasis of oral squamous cell carcinoma (OSCC).^29^ Here, we found that the high expression of MHC class Ⅰ molecules by *IFIT1*^+^ tumor cells enhances the cytotoxicity of CD8^+^ T cells and promotes the increased expression of IFN-γ and granzyme B in CD8^+^ T cells. These findings collectively highlight the crucial role of *CXCL10* and *IFIT1* in shaping the TME and influencing the biological behavior of HGSOC.

The TME in HGSOC is likewise inherently complex, comprising various cell types including immune cells, stromal cells, and other epithelial populations. The presence of *CXCL10*^+^ and *IFIT1*^+^ epithelial cells may shape the TME by influencing the immune landscape. Our subsequent analysis confirmed that *IFIT1* is not only closely related to the prognosis of HGSOC but may also affect the expression of MHC class Ⅰ molecules, activate CD8^+^ T cells, and thereby play a crucial role in regulating the immune microenvironment of tumor occurrence and development. Therefore, understanding the specific characteristics of *IFIT1*^+^ expressing cells, including their origins and interactions with other tumor-associated cells, is crucial for developing targeted therapies that address the multifaceted nature of HGSOC.

### Limitations of the study

This work is subject to several limitations. The cohort size, while informative, is constrained and might not encompass the full spectrum of clinical and molecular heterogeneities present in HGSOC. Additionally, the scRNA-seq approach is prone to technical limitations, including transcript dropouts, batch effects, and biases potentially arising from tissue dissociation, all of which may influence the accurate representation of cellular populations. Our analysis also does not incorporate the spatial organization of the TME and offers a static view that cannot track temporal tumor evolution. While computational analyses indicate possible therapeutic targets and immune evasion pathways, these insights necessitate functional confirmation using *in vitro* or *in vivo* experimental systems. Future studies incorporating multiomics data, such as proteomic or epigenomic layers, would further elucidate the underlying mechanisms.

## Resource availability

### Lead contact

Further information and requests for resources and reagents should be directed to and will be fulfilled by the lead contact, Wu Wenjuan (wjw1010@foxmail.com).

### Materials availability

Main data are available in the main text and supplemental information. Other materials and procedures will be made available upon request.

### Data and code availability

• This paper reported in this paper are available in deposited data in the key resources table.
• All original code can be found at https://zenodo.org/records/18309045.
• Any additional information required to reanalyze the data reported in this paper is available from the lead contact upon request.

## Acknowledgments

This research was funded by 10.13039/100007452Wu Jieping Medical Foundation (grant number 320.6750.2024-13-20) and Plan on enhancing scientific research in GMU and Guangdong Yiyang Healthcare Charity Foundation (grant number JZ2024042).

## Author contributions

Conceptualization and design, C.G. and W.W.; data acquisition, C.G., X.L., A.P., and Q.L.; data analysis and interpretation, C.G., X.L., A.P., D.Z., and W.W; statistical analysis, C.G., X.L., D.Z., L.C., and W.W.; funding acquisition, C.G. and W.W.; writing – original draft, C.G., X.L., A.P., D.Z., and W.W. All authors have read and approved the final manuscript.

## Declaration of interests

The authors declare that they have no competing interests.

## STAR★Methods

### Key resources table

**Table:** 

REAGENT or RESOURCE	SOURCE	IDENTIFIER
**Antibodies**		

IFIT1 antibody	Absin	Cat# abs149003 RRID: N/A
CD8 antibody	Abcam	Cat# ab237709 RRID: AB_2892677
B2M antibody	Abcam	Cat# ab215889 RRID: N/A
Pan-CK antibody	Abcam	Cat# ab215838 RRID: AB_2922672
CD133 antibody	Cell Signaling Technology	Cat# 60577 RRID: AB_2799590
IFIT1 antibody	Cell Signaling Technology	Cat# 14769 RRID: AB_2783869

**Biological samples**		

Human ovarian serous cancer tissues	the Affiliated Cancer Hospital, Guangzhou Medical University	–

**Chemicals, peptides, and recombinant proteins**		

FBS	Gibco	Cat #A5256801
HEPES	Life Technologies	Cat #15630080
CD3/CD28 Dynabeads	Life Technologies	Cat #11456D
IL-7	Biolegend	Cat #577802
IL-15	Biolegend	Cat #566302
IL-2	Biolegend	Cat #575402
Collagenase	Sigma-Aldrich	Cat# C9407
Percoll	Sigma-Aldrich	Cat# 1644
DNAse I	STEMCELL Technologies	Cat # 07900
Lympholyte Cell Separation Media	Cedarlane Laboratories	Cat. No. CL5035
RBC lysis buffer	ThermoFisher Scientific	Cat # 50-112-9751
FACS buffer	BD Biosciences	Cat# 554656
Fixable Viability Stain 510	BD Biosciences	Cat# 564406

**Critical commercial assays**		

OpalTM 4 Color Manual IHC Kit	Akoya Biosciences	Cat# NEL810001KT
FITC Annexin V Apoptosis Detection Kit Ⅰ	BD Biosciences	Cat# 556547
Human IFN-gamma Quantikine QuicKit ELISA	R&D Systems	Cat# QK285
Human Granzyme B DuoSet ELISA	R&D Systems	Cat# DY2906-05

**Deposited data**		

scRNA-seq dataset of ovarian cancer	Xu et al.^17^	GEO: GSE184880
scRNA-seq dataset of ovarian cancer	Loret et al.^30^	GEO: GSE201047
scRNA-seq dataset of ovarian cancer	Zhang et al.^31^	GEO: GSE165897
scRNA-seq dataset of colorectal cancer	Zhang et al.^32^	GEO: GSE146771
scRNA-seq dataset of bladder cancer	Wang et al.^33^	GEO: GSE130001

**Experimental models: Cell lines**		

C57BL/6J	GemPharmatech Co., Ltd.	No: N000013

**Software and algorithms**		

GraphPad Prism 10	GraphPad Prism	https://www.graphpad.com
FlowJo	TreeStar	https://www.flowjo.com/solutions/flowjo
Seurat V4.4.0	CRAN	https://cran.r-project.org/web/packages/Seurat/index.html
Philentropy V0.8.0	CRAN	https://cran.r-project.org/web/packages/philentropy/index.html
Infercnv V1.20.0	Github	https://github.com/broadinstitute/infercnv
clusterProfiler V4.8.2	Bioconductor	https://bioconductor.org/packages//release/bioc/html/clusterProfiler.html
org.Hs.eg.db V3.17.0	Bioconductor	https://bioconductor.org/packages//2.7/data/annotation/html/org.Hs.eg.db.html
SMDIC V0.1.5	CRAN	https://cran.r-project.org/web/packages/SMDIC/index.html
CellChat V2.1.2	Github	https://github.com/jinworks/CellChat

### Experimental model and study participant details

#### Study cohorts

Paraffin-embedded tissues from 50 ovarian serous cancer patients (2014-2025) were retrospectively collected from the GZ Tumor BioBank, with waived consent for anonymized data. Prospectively, fresh tumor tissues and peripheral blood were obtained from ovarian cancer patients and healthy volunteers, respectively (Table S1). All patients received cytoreductive surgery plus paclitaxel/carboplatin chemotherapy. The retrospective study (GYZL-2024-KY35-02) and prospective collection (GYZL-KY-2025089-01) were both approved by the Institutional Review Board (IRB) of Guangzhou Institute of Cancer Research, the Affiliated Cancer Hospital, Guangzhou Medical University, with written informed consent obtained for all prospective participants.

### Method details

#### Data acquisition

This study includes three cohorts consisting of primary and metastatic HGSOC (GSE184880^17^ [n = 7], GSE201047^30^ [n = 3] and GSE165897^31^ [n = 11]), as well as two cohorts consisting, respectively, of 10 patients with colon cancer (GSE146771^32^) and 2 patients with bladder cancer (GSE130001^34^). These datasets, containing corresponding scRNA-seq data, were extracted from the GEO database (https://www.ncbi.nlm.nih.gov/geo/). Detailed information of the included samples is provided in Tables S2 and S3.

#### scRNA-seq data processing

R software with the Seurat (v.4.4.0) package was used to perform quality control (QC) on 17 HGSOC tissue samples (10 primary, 3 ascites metastasis, 2 peritoneal metastasis, and 2 omental metastasis) from 10 patients. To exclude low-quality cells and lowly expressed genes, the following criteria were applied: (1) each gene must be expressed in at least 3 cells; (2) each cell must have >1,000 counts and gene counts between 200 and 8000; (3) the proportion of mitochondrial genes per cell must be less than 20%. To remove batch effects resulting from pooling different datasets, we performed batch effect correction on the post-QC data. Principal component analysis was performed on the scaled data to derive the top 25 principal components for subsequent analyses. The t-SNE algorithm was used to visualize the data. The k-nearest neighbors (kNN) algorithm and ‘FindClus-ters’ function were used for determining cell clusters. The ‘FindMarkers’ function in the Seurat package was used to find marker genes for each cluster by setting the parameters to (avg_log2FC>0.25, p_val_adj<0.05, min.pct = 0.1, only. pos = TRUE). Finally, the an-notation of each cell cluster was confirmed by the expression of canonical marker genes.

#### Comparison of major cell types across different samples

The Jensen-Shannon divergence (JSD) metric was employed to analyze differences between cell types. First, we calculated the proportions of major cell types in each sample. To compute the differences in cell composition between the samples, the JSD score was calculated using the R package philentropy.

#### Inferred copy number variation analysis of dominant cell types

Inferred copy number variation (CNV) analysis was conducted using the inferCNV (v1.20.0) (cutoff=0.1) R package. All cell types except epithelial cells were taken as reference. This approach allowed us to compare the differences in copy number between epithelial cells and other cell types. We evaluated the significance of these differences with the rank-sum test.

#### Identification of cell subpopulations

Cell subpopulations within the major cell types were defined through identification of highly variable genes, dimensionality reduction, and clustering. Differentially ex-pressed genes (DEGs) in each cell subset were identified using the ‘FindAllMarkers’ function (min. pct = 0.25,logFC. threshold = 0.25, only. pos = TRUE) in Seurat. Epithelial cell subpopulations were annotated using hypergeometric enrichment test instead of known markers from the literature.

#### Functional and pathway enrichment analyses

To explore the functional relevance of the candidate cell clusters, we performed functional annotation enrichment analysis of upregulated DEGs with Gene Ontology and KEGG using the R packages clusterProfiler (4.8.2) and org.Hs.eg.db (3.17.0). Enriched terms with adjusted P < 0.05 were considered statistically significant.

#### Analysis of tumor-infiltrating immune cells

Bulk RNA-seq data from the TCGA-ovarian serous cystadenocarcinoma (TCGA-OV) dataset was used for estimations of immune cell type proportions in candidate cell clusters and assessments of clinical significance. Probes were collapsed by gene symbols and expression levels were averaged over multiple probes mapped to the same gene symbol. Because expression profiles were log2 (FPKM+1) transformed, we first reverted this transformation. Then, CIBERSORT using the ‘exp2cell’ function from SMDIC (0.1.5) was applied to estimate the abundance of 22 immune cell types. Correlations were conducted using the ‘cor( )’ function from the stats package, and visualized using the cor.mtest function from the corrplot package (v.0.92). Significant (P < 0.05) genes or cell pairs were selected for subsequent analysis.

#### Single-cell trajectory and cell-cell interaction analyses

The ‘plot_cell_trajectory’ function in Monocle 2^35^ was applied to generate a pseudotime developmental trajectory of different endothelial cell clusters based on gene expression patterns. The CellChat R package (v2.1.2)^36^ was used for cell-cell communication analysis, to assess differences in the number and strength of inferred interactions. Interactions among key cell types were selected for further analysis.

#### Multiplex immunohistochemistry

A 6-color panel was designed for multiplex immunohistochemistry (mIHC). The mIHC assay was conducted by RayBiotech Guangzhou Co., Ltd. using FFPE tumor tissues. The following antibodies were utilized in the assay: IFIT1 (Absin, No. abs149003), CD3 (ABcam, No. ab16669), CD8 (Abcam, No. ab237709), B2M (Abcam, No. ab215889), Pan-CK (Abcam, No. ab215838), and then tyramide signal amplification visualization was performed using OpalTM 7 Color Manual IHC Kit (NEL811001KT, Akoya). Digital images were scanned and visualized in SlideViewer software (Akoya).

#### Flow cytometry

For the CD8^+^ T cell cytotoxicity analysis, peripheral blood was collected from healthy volunteers. Peripheral blood samples were collected from healthy volunteers, and PBMCs were isolated via density gradient centrifugation with Human Lymphocyte Isolation Solution (GOONIE, 100-306) using a floor-type centrifuge (SCRNSPEEO, 1736R). After isolation, PBMCs were resuspended in RPMI-1640 medium (VivaCell) supplemented with 10% FBS. CD8^+^ T cells were purified from PBMCs through negative magnetic sorting (Thermo Fisher Scientific, 11348D) and activated with CD3/CD28 Dyna-beads (1:1 ratio of cells: beads) 2.5 ng/ml IL-7, 50 ng/ml IL-15, and 2 ng/ml IL-2 for 2 days.

For tumor tissue processing, samples were digested using DNase I (0.5 mg/ml, Sigma) and collagenase (1 mg/ml, Sigma). The resulting cell suspension was filtered through a 70-μm cell strainer (absin, abs7232) and then washed with PBS. Viable cells were labeled with Fixable Viability Stain 510 (BD Biosciences, 564406). And the cells were incubated antibodies as anti-CD133 (Cell Signaling Technology, #60577) and anti-IFIT1 (Cell Signaling Technology, #14769).

The stimulated CD8^+^ T cells co-cultured with the CD133^+^ IFIT1^+^ or CD133^+^ IFIT1^-^ cells at an effector/target ratio of 10:1 for another 48 h at 37°C. Then the harvested cells were detected with FITC Annexin V Apoptosis Detection Kit Ⅰ (BD Biosciences, 556547) and stained for Annexin V 5 U/test, and cell apoptosis was detected.

#### ELISA assay

The conditioned medium (CM) of co-cultured CD8^+^ T cells and CD133^+^ IFIT1^+^ or CD133^+^ IFIT1^-^ cells were analyzed to measure IFN-γ or GZMB levels using ELISA kits (R&D Systems, QK285, DY2906-05). The assays were performed in triplicates according to the manufacturer’s instructions.

### Quantification and statistical analysis

All statistical analyses are detailed in this section and in the corresponding figure legends. The figure legends explicitly state the specific statistical test used, the exact value of n, and the measures of center and dispersion. Bioinformatics Analyses: Software, algorithms, and key parameters for all computational analyses are described in the relevant subsections of the STAR Methods. Experimental Validation: Statistical methods for data from experiments are described here. All experiments were performed with three independent biological replicates. Data were analyzed using GraphPad Prism (version 10.0). For comparisons between two groups with similar variance, unpaired two-tailed Student’s t-tests were employed. Statistical significance is denoted as follows: ∗p < 0.05, ∗∗p < 0.01. A p-value of less than 0.05 was considered statistically significant.

## Supplemental information

Document S1. Figures S1–S7 and Tables S2, S3, and S6

Table S5. Enrichment analysis of different epithelial cell clusters, related to Figures 3C and 3D
