# Supplementary material for: Single-cell transcriptomic analysis reveals intra-tumoral heterogeneity and immunotherapy strategies in high-grade serous ovarian cancer
Source: iScience. 2026 Mar 6;29(4):115266. doi: 10.1016/j.isci.2026.115266 (PMC13018870; doi:10.1016/j.isci.2026.115266)
Supplement: Document S1. Figures S1–S7 and Tables S2, S3, and S6 [file mmc1.pdf]

iScience, Volume *29*

Supplemental information

Single-cell transcriptomic analysis reveals intra-tumoral heterogeneity and immunotherapy strategies in high-grade serous ovarian cancer

Chunhui Gao, Xiaogang Lv, Anqi Pan, Lipai Chen, Qianqian Liu, Donghui Zhang, and Wenjuan Wu

Supplementary Figures

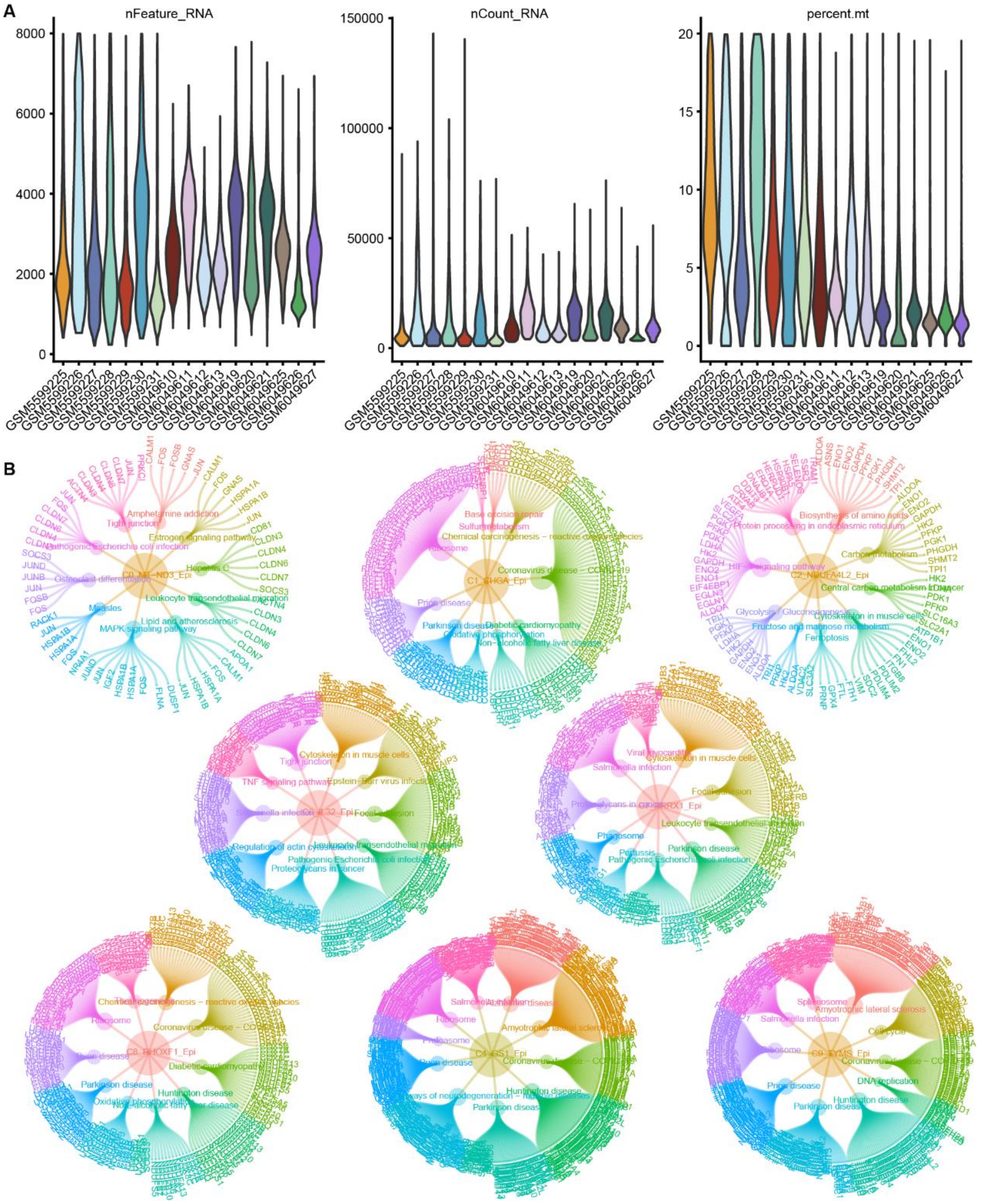

Figure S1. Quality control and pathway enrichment analysis, related to Figure 1 and 2. (A) Information for each sample after quality control. (B) Top 10 pathways in different epithelial cell clusters by KEGG pathway enrichment analysis.

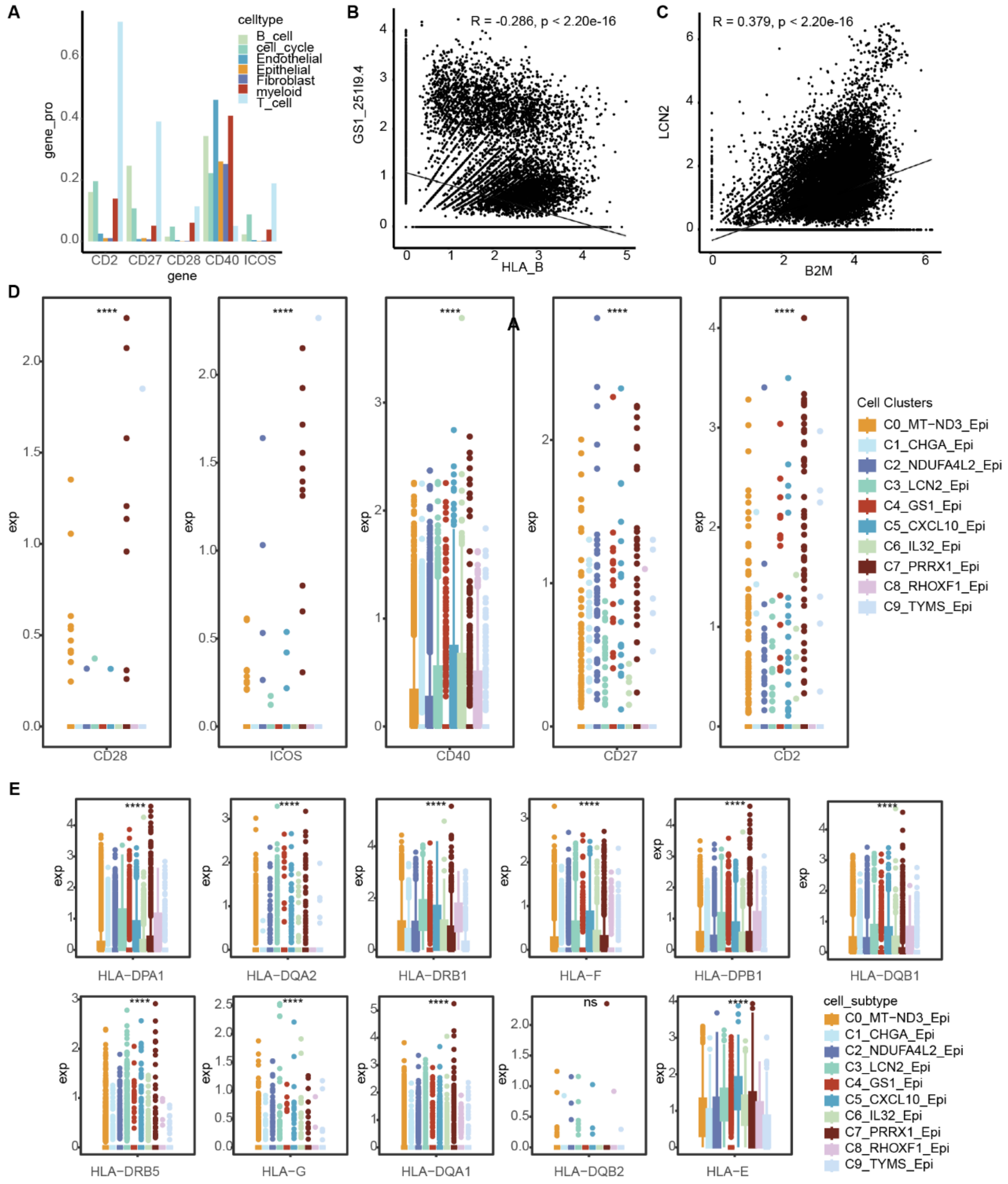

Figure S2. Immunogenicity analysis, related to Figure 1G-H, 2D and 3. (A) Proportions of co-stimulatory molecules in different cells. (B) Scatter plot of the gene pairs with the highest negative correlation, where each point represents a sample. (C) Scatter plot of the gene pairs with the highest positive correlation, where each point represents a sample. (D) Expression levels of co-stimulatory molecules in 10 cell clusters. (E) Expression level of MHC molecules in different cell clusters.

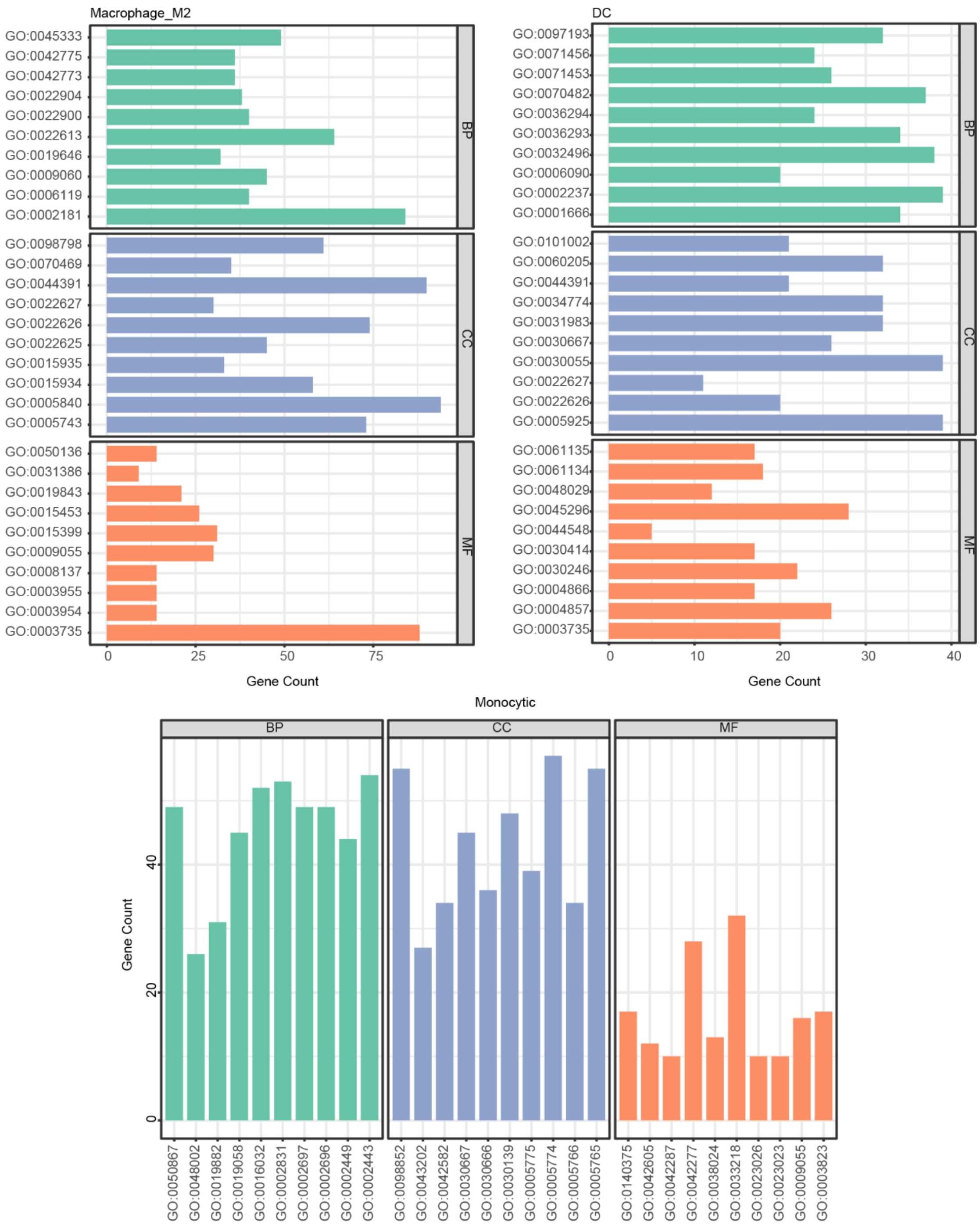

Figure S3. GO annotation for M2 macrophages, dendritic cells, and monocytes, related to Figure 4E and 4F.

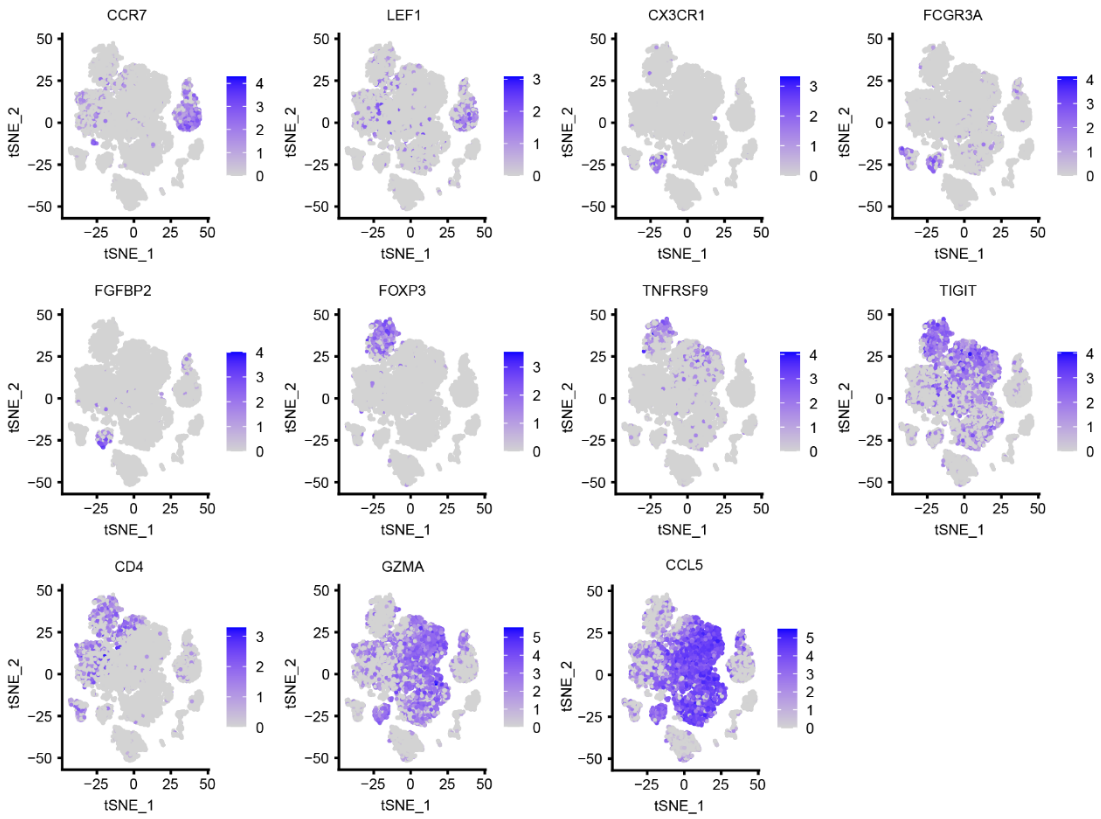

Figure S4. t-SNE plot of marker gene expression for T cell subpopulations, related to Figure 5.

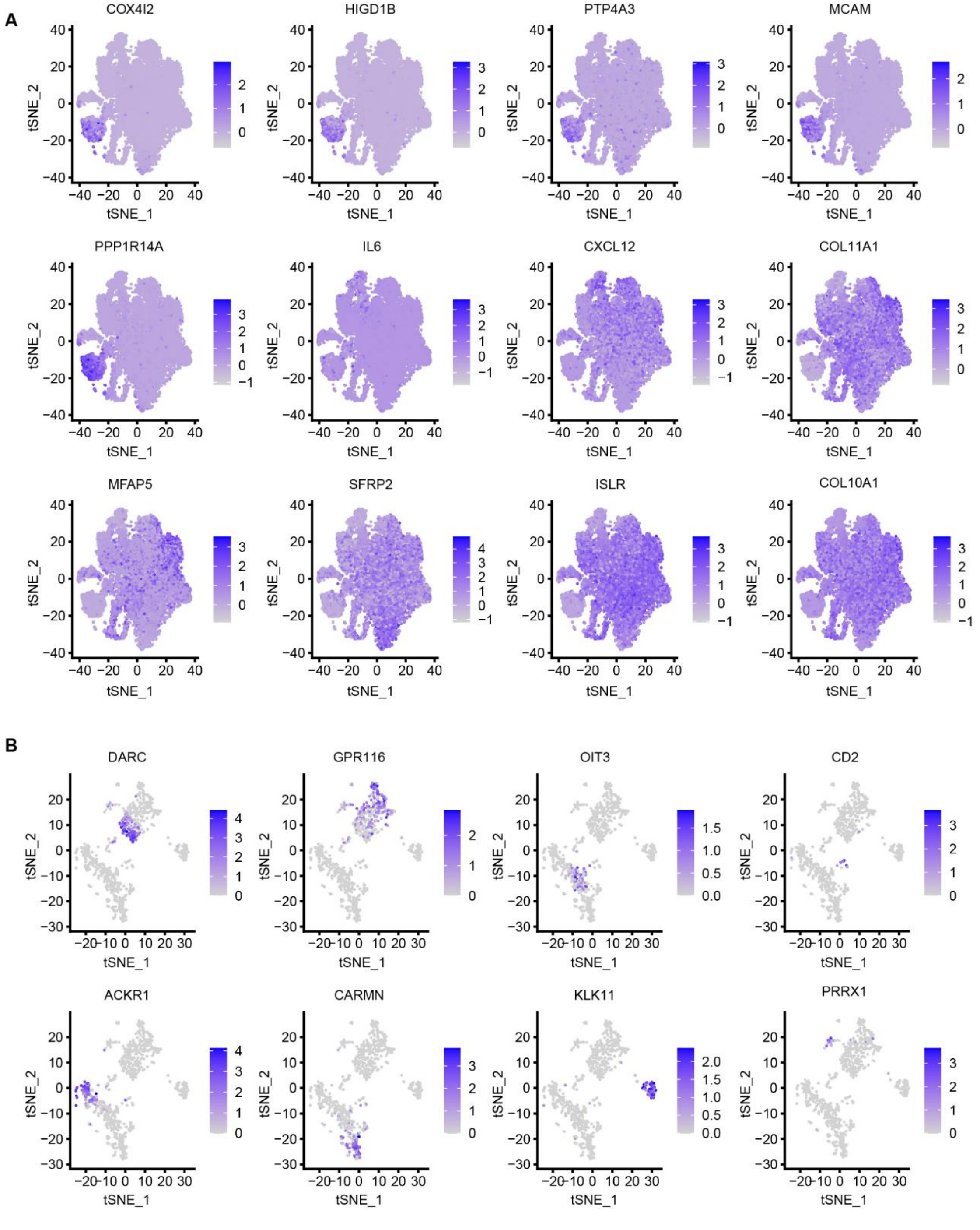

Figure S5. t-SNE plots of marker gene expression, related to Figure 6A and 6D. (A) Expression levels of classic marker genes for fibroblasts. (B) Expression levels of the top-ranking gene in different clusters of endothelial cells.

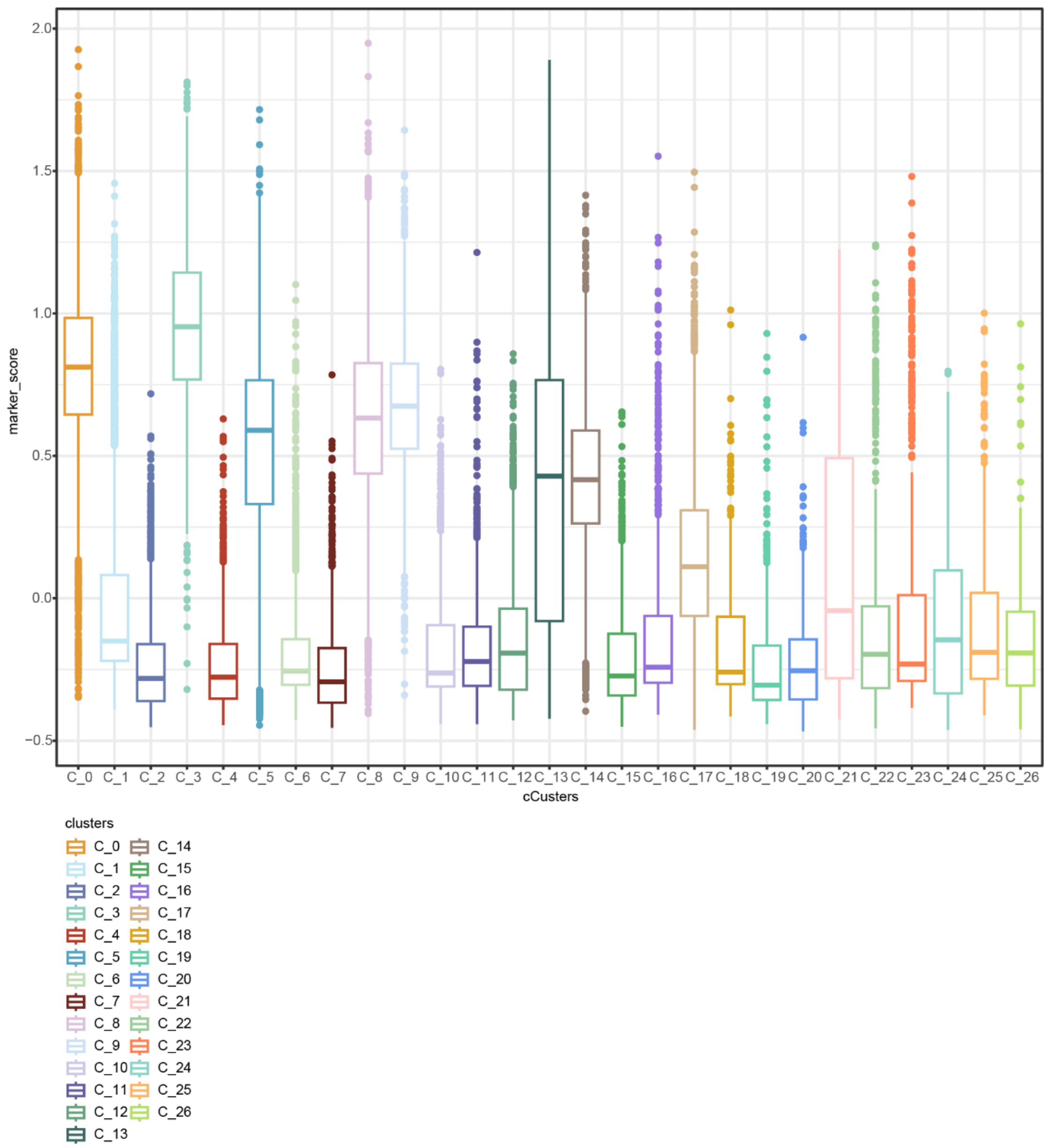

Figure S6. Scoring for different cell clusters based on tumor-associated markers, related to Figure 8B.

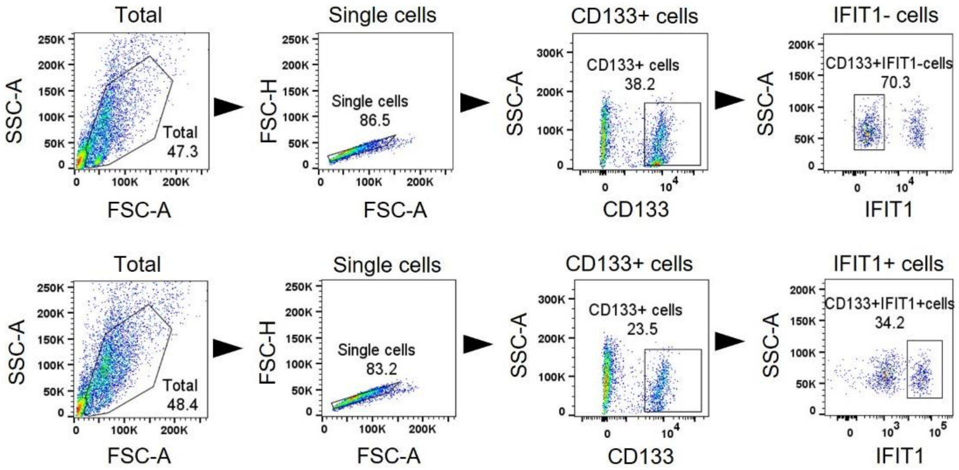

Figure S7. Flow cytometry gating strategy and proportion for the IFIT1^{-} or IFIT1^{+} population is illustrated. Representative plots are shown, related to Figure 8J.

Supplementary Table 2. Sample information from HGSOC, bladder cancer and colon cancer GEO datasets, related to Figure 1.

Dataset ID	Platform	No. Of Samples
GSE184880	GPL24676	7
GSE201047	GPL20301	10
GSE165897	GPL16791; GPL20301; GPL24676	11
GSE146771	GPL20301	10
GSE130001	GPL16791	2

Supplementary Table 3. Sample Information Included in the HGSOC Dataset, related to Figure 1.

Sample	Dataset ID	Source
GSM5599225	GSE184880	Primary
GSM5599226	GSE184880	Primary
GSM5599227	GSE184880	Primary
GSM5599228	GSE184880	Primary
GSM5599229	GSE184880	Primary
GSM5599230	GSE184880	Primary
GSM5599231	GSE184880	Primary
GSM6049610	GSE201047	Primary_Met
GSM6049611	GSE201047	Met_ascites
GSM6049612	GSE201047	Met_PER
GSM6049613	GSE201047	Met_OM
GSM6049619	GSE201047	Primary_Met
GSM6049620	GSE201047	Met_ascites
GSM6049621	GSE201047	Met_OM
GSM6049625	GSE201047	Primary_Met
GSM6049626	GSE201047	Met_ascites
GSM6049627	GSE201047	Met_PER

Supplementary Table 6. Marker genes for extracellular vesicle (EV) were obtained from the literature, related to Figure 8C and 8D.

Biomarker	Source
TSG101	PMID: 29384376
ALIX (PDCD6IP)	PMID: 29384376
CD9	PMID: 29384376
CD63	PMID: 29384376
CD81	PMID: 29384376
